# Changes in corticospinal excitability and the direction of evoked movements during motor preparation: A TMS study

**DOI:** 10.1186/1471-2202-9-51

**Published:** 2008-06-17

**Authors:** Gijs van Elswijk, Willemijn D Schot, Dick F Stegeman, Sebastiaan Overeem

**Affiliations:** 1 Department of Clinical Neurophysiology, Radboud University Nijmegen Medical Centre, The Netherlands; 2 F.C. Donders Centre for Cognitive Neuroimaging, Radboud University Nijmegen, The Netherlands; 3 Faculty of Human Movement Sciences, VU University Amsterdam, The Netherlands

## Abstract

**Background:**

Preparation of the direction of a forthcoming movement has a particularly strong influence on both reaction times and neuronal activity in the primate motor cortex. Here, we aimed to find direct neurophysiologic evidence for the preparation of movement direction in humans. We used single-pulse transcranial magnetic stimulation (TMS) to evoke isolated thumb-movements, of which the direction can be modulated experimentally, for example by training or by motor tasks. Sixteen healthy subjects performed brisk concentric voluntary thumb movements during a reaction time task in which the required movement direction was precued. We assessed whether preparation for the thumb movement lead to changes in the direction of TMS-evoked movements and to changes in amplitudes of motor-evoked potentials (MEPs) from the hand muscles.

**Results:**

When the required movement direction was precued early in the preparatory interval, reaction times were 50 ms faster than when precued at the end of the preparatory interval. Over time, the direction of the TMS-evoked thumb movements became increasingly variable, but it did not turn towards the precued direction. MEPs from the thumb muscle (agonist) were differentially modulated by the direction of the precue, but only in the late phase of the preparatory interval and thereafter. MEPs from the index finger muscle did not depend on the precued direction and progressively decreased during the preparatory interval.

**Conclusion:**

Our data show that the human corticospinal movement representation undergoes progressive changes during motor preparation. These changes are accompanied by inhibitory changes in corticospinal excitability, which are muscle specific and depend on the prepared movement direction. This inhibition might indicate a corticospinal braking mechanism that counteracts any preparatory motor activation.

## Background

Many attributes of voluntary or instructed movements are prepared in advance, in order to facilitate efficient execution. Such facilitation may for example result in enhanced accuracy and shortened reaction times. Preparation of movement direction may already begin about 100 ms after presentation of a directional cue [1]. Moreover, providing prior information (i.e. precueing) about the direction of an upcoming movement results in a strong reduction in reaction times [2,3].

On the neurophysiologic level, directional coding has been studied extensively in behaving animals. The activity of single neurons in the motor cortex is gradually modulated by movement direction [4,5]. The direction of any particular movement can therefore be encoded across a population of motor cortical cells. Neuronal coding of direction has been reported not only to occur shortly before or during the execution of movements but also during preparatory intervals, several hundreds of milliseconds before the onset of actual movement [6-10]. Furthermore, preparatory activity of direction-selective neurons in monkey motor cortex can predict the direction and the reaction time of a subsequent movement on a trial-to-trial basis [6,11]. Thus it seems that prior information about movement direction facilitates reaction time through pre-activation of neuronal output systems.

Here we aimed to find neurophysiologic evidence for the preparation of movement direction in humans. Although it is virtually impossible to measure directional coding of individual neurons in healthy humans, on a macroscopic level the representation of movement direction in motor cortex can be investigated with transcranial magnetic stimulation (TMS). Classen and colleagues introduced the use of TMS-evoked thumb movements to reveal changes in motor-cortical movement representations [12]. By assessing the direction of TMS-evoked thumb movements they showed that these movement representations can be modulated experimentally [12-14]. TMS-evoked movements have also been applied in reaction tasks to show that their directions starts to follow the intended movement well before the start of the voluntary response, but these studies focussed on the period *after *the response signal (i.e. the response interval) [15,16]. In this study, we assessed whether purely preparatory processes influence the cortical movement representation and if the direction of an intended movement may already be determined *prior *to the response signal, namely the preparatory interval.

The brief muscle twitches underlying TMS-evoked movements can be recorded electromyographically as so called motor-evoked potentials (MEP). Their amplitudes are a measure of corticospinal excitability. Several earlier studies have made clear that corticospinal excitability of movement agonists increases during the last 100 ms of the response interval [15-21]. Recent TMS research has shown that if a specific movement can be prepared, corticospinal excitability of the movement agonists may also increase during the preparatory interval [22-24]. If preparation of a specific movement direction is reflected by the kinematics of TMS-evoked movements, it should correspondingly modulate corticospinal excitability. During preparation of a particular direction, the agonists are expected to be facilitated. However, there is also accumulating evidence for an important role of corticospinal inhibition in motor preparation [25-29]. This inhibition seems to be related to the estimation of the timing of the response signal rather than to preparation of a specific response, as it is not specific to movement agonists [28]. Inhibition associated with time preparation appears to be aimed at spinal circuits whereas response specific preparation most likely occurs at the cortical level [26,30,31].

**Figure 1:**
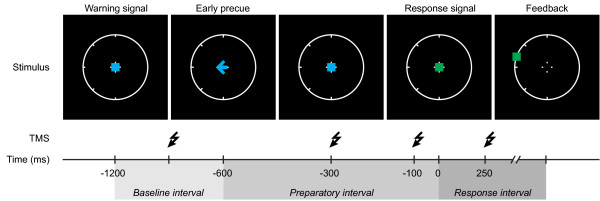
**Schematic representation of the events in a go trial with an early precue**. In each trial a warning signal and a response signal were presented. The precue was presented at either 600 ms (early precue) or 100 ms (late precue) before the response signal. A response was required in go trials only (green coloured response signal; 80% probability). In a random 280 (~78%) of the 360 early-precue trials, TMS was applied at either 900, 300, or 100 ms before, or 250 ms after the response signal. A trial can be divided into three epochs. The epoch before the precue is termed the baseline interval; the epoch between precue and response signal is termed the preparatory interval; the epoch after the response signal is termed the response interval. These epochs are marked using shades of grey, also in the following figures.

We sought to elucidate whether prior specification of the required direction of an upcoming movement is associated with changes in the corticospinal output (not only changes in TMS-evoked movements but also associated changes in corticospinal excitability) during the preparatory interval preceding that movement. Our subjects performed a motor preparation task that involved brisk concentric thumb movements. The required movement direction was instructed via a precue (Figure 1). In order to modulate the amount of preparation that could be attained, the precue either was presented relatively late in the preparatory interval or relatively early in the preparatory interval. Compared to a late precue, an early precue provided an additional period of 500 milliseconds where movement direction could be prepared, and was hence expected to yield a shorter reaction time. In trials with an early precue, we used TMS to determine 1) whether the directional precue modulates the direction of TMS-evoked thumb movements; 2) how these changes relate to changes in corticospinal excitability, as reflected by MEPs from hand muscles.

## Results

In total, 13% of all trials were discarded because subjects made errors, and/or pretrigger electromyographic (EMG) activity was detected.

### Voluntary movements

**Figure 2:**
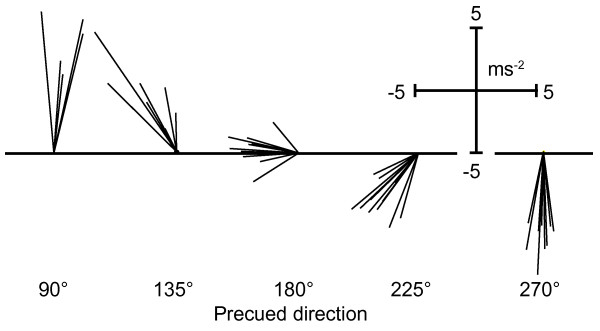
**Example acceleration vectors of voluntary thumb movements**. First-peak acceleration vectors from early-precue trials without TMS. Data from one subject. The plot shows that each of the five precues induced a thumb movement in a different direction, with little overlap between movements in response to different precues.

**Figure 3:**
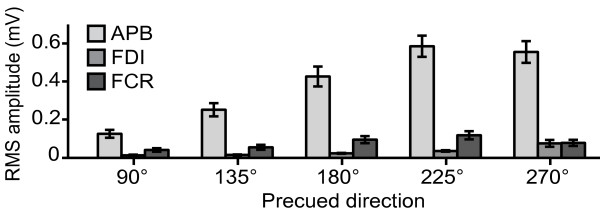
**Muscle activity during voluntary thumb movements**. Mean (± *SE*) root-mean-square amplitude across subjects (*N*= 16) of the EMG activity during the initial 150 ms of the voluntary response, for each of the five precues and each of the three EMG channels.

Examples of voluntary thumb movements of one of the subjects are shown in Figure 2. On average (*N *= 16), the movement direction deviated 15.6 ± 2.0 degrees from the precued direction. The ANOVA [32] showed that average movement directions were significantly different between all five precue conditions [*F*(4,60) = 407.13, *p *< 0.001; all pair wise comparisons *p *< 0.001]. Thus, subjects were able to accurately move their thumb in all five directions, with little overlap between movements in response to different precues. Figure 3 shows for each muscle the average RMS amplitudes across subjects in the five different movement directions. During movement, the thumb muscle (measured with electrodes over the abductor pollicis brevis, therefore labelled 'APB' throughout the paper) displayed the largest EMG amplitudes. Furthermore, the thumb muscles were most strongly activated during movement directions of 180°, 225°, and 270° (corresponding to abduction/flexion). The EMG amplitudes of the index finger muscle (measured with electrodes over the first dorsal interosseus, labelled 'FDI') and the wrist flexor (measured with electrodes over the flexor carpi radialis, labelled 'FCR') were much smaller than the amplitudes of the thumb muscle indicating little involvement in any movement direction.

**Figure 4:**
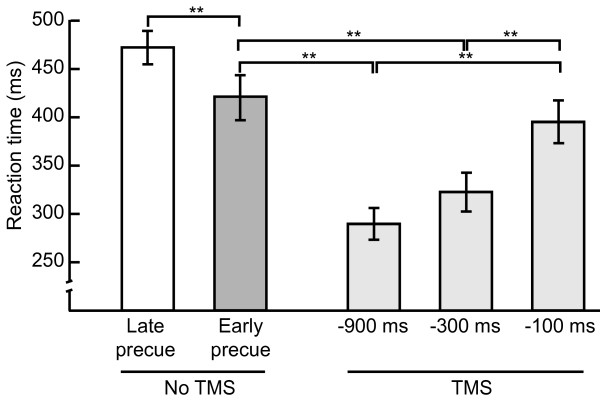
**Reaction time**. Mean (± *SE*) reaction time (RT) across subjects (*N *= 16), as a function of precue onset and stimulation time. The RT difference between the late- and the early-precue condition strongly suggests that at least parts of the thumb movement were programmed before the response signal occurred. The early-precue trials without TMS were also compared to early-precue trials with TMS. The post-hoc comparisons showed that TMS reduced the reaction times even further, and that this reduction was stronger the earlier the TMS pulse was applied. **p < 0.01.

The average RT of the early-precue trials without TMS was 422 ± 23 ms (Figure 4). This was significantly faster than the late-precue trials that had an average RT of 472 ± 17 ms [*t(15) *= 3.03, *p *< 0.01].

There was a significant reduction in RT when a TMS pulse was applied during the preparatory interval [*F*(3,45) = 23.45, *p *< 0.001] (see Figure 4). Post-hoc comparisons showed that a TMS pulse at -900 ms or at -300 ms before the response signal significantly reduced RT compared to no-TMS or to TMS at -100 ms before the response signal (pairwise significance levels shown in Figure 4).

### TMS-evoked movements

**Figure 5:**
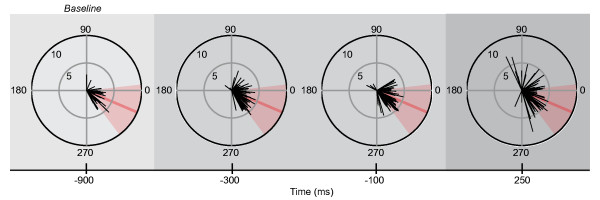
**Example acceleration vectors of TMS-evoked movements**. First-peak acceleration vectors of TMS-evoked movements of one of the subjects at the four stimulation times, for all precues combined. Black lines show acceleration vectors (magnitude-direction) of individual movements. The thick red line depicts the average angle of movements evoked during the baseline interval (-900 ms). This angle was used to define the baseline zone (± 30°), which is marked in light red. Baseline, preparatory, and response intervals are marked by light, medium, or dark grey background, respectively (see Figure 1). The plots show a decrease in the proportion of TMS-evoked movements that fell into the baseline zone, at the end of the preparatory interval. This proportion further decreased during the response interval.

**Figure 6:**
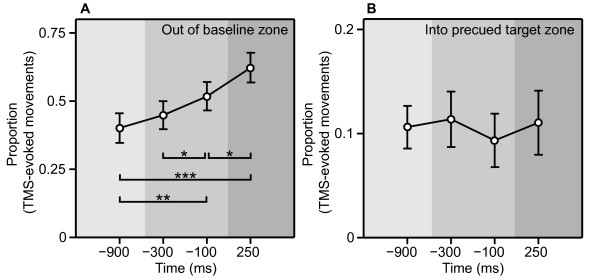
**TMS-evoked movements**. Mean proportion (± *SE*) of TMS-evoked movements as a function of time that fell (*A*) outside the ± 30° zone around the baseline direction, or (*B*) inside the ± 30° zone around the precue. Baseline, preparatory, and response intervals are marked by light, medium, or dark grey background, respectively (see Figure 1). Compared to the baseline interval, increasingly more TMS-evoked movements fell outside the baseline zone. Thus, the angles of the TMS-evoked movements were modulated over time, indicating changes in the thumb movement cortical representation. However, there was no increase in the number of TMS-evoked movements that fell into the ± 30° zone around the precued direction. *p < 0.05, **p < 0.01.

Examples of TMS-evoked thumb movements in a single subject, obtained at the four stimulation times are plotted in Figure 5. Analysis of the group results showed that over time a significantly increasing proportion of TMS-evoked movements fell outside the baseline zone (a ± 30° window centred on the average movement direction during baseline; see Figure 6A) [*F*(3,45) = 14.74, *p *< 0.001]. Before precue onset, 40% ± 5.4% of the TMS-evoked movements fell outside this baseline zone and this progressively increased to 62% ± 5.5%. Post-hoc tests revealed a significant change in the proportion already within the preparatory interval, namely the increase from 45% ± 5.3% to 52% ± 5.3% between 300 and 100 ms before the response signal [*p *< 0.05]. This proportion further increased to 62% ± 5.5% during the response interval [*p *< 0.05]. Compared to the baseline, the proportion of TMS-evoked movements falling outside the baseline zone was increased at -100 and 250 ms [*p *< 0.01 and *p *< 0.001, respectively].

Further analyses showed that there were no changes in the proportion of TMS-evoked movements falling within the target zone (a ± 30° window centred on the precued direction) [*F*(3,45) = 0.34, n.s.] (See Figure 6B). This result was surprising, as we expected an increase in the TMS-evoked movements at least during the phase after the response signal (cf. [15]). Therefore, we conducted further analyses. An increase in the proportion of movements in the precued target zone might be revealed for precued directions near the baseline thumb movement direction. To this end, we divided the precued directions into two categories: a) precued directions with a small deviation (≤ 90°) from the baseline direction, b) precued directions with a large deviation (> 90°) from the baseline direction. Similar to the initial analysis, we then analysed the time-dependent change in the proportion of TMS-evoked movements falling within the target zone for each of these two categories separately. As expected, for targets near the baseline direction a larger proportion fell into the target zone than for targets far from the baseline [17.4 ± 3.4 % vs. 4.5 ± 0.9 %; *F*(3,45) = 14.92, *p *< 0.01]. However, the ANOVAs did again not reveal any significant temporal modulation of the proportion of TMS-evoked movements in the target zone, neither for targets near the baseline direction [*F*(3,45) = 1.18, n.s.], nor for targets far from the baseline direction [*F*(3,45) = 1.74, n.s.].

Thus after precueing, the direction of TMS-evoked thumb movements became increasingly less consistent, but we found no indication for a specific implementation of the precued direction.

### Motor-evoked potentials

**Figure 7:**
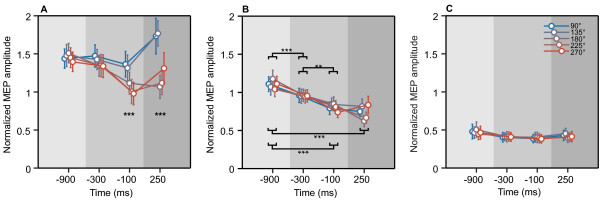
**Motor-evoked potentials**. Mean (± *SE*) normalised motor-evoked potential (MEP) amplitudes (*N *= 16) as a function of time and precued direction, for the abductor pollicis brevis (*A*), first dorsal interosseus (*B*), and flexor carpi radialis (*C*) of the moving hand. Baseline, preparatory, and response intervals are marked by light, medium, or dark grey background, respectively (see Figure 1). In (*A*) the stars denote the significance level of omnibus *F*-test (one-way ANOVA), the corresponding post-hoc comparisons are specified in Table 1. In (*B*) the stars denote the significance levels of post-hoc comparisons. *p < 0.05, **p < 0.01, and ***p < 0.001. The MEP amplitudes in the APB and the FDI were significantly modulated during the preparatory and the response interval. For the APB, there was a significant effect of the precued direction at -100 ms and 250 ms (see Table 1). The MEPs of the FDI were not differentially modulated by the precue, but generally decreased over time. The MEP amplitudes in the FCR did not change significantly.

The initial three-way ANOVA showed significant main effects of muscle [*F*(2,30) = 20.39, *p *< 0.001], precue [*F*(4,60) = 9.88, *p *< 0.001], and time [*F*(3,45) = 9.88, *p *< 0.01]. All interactions were significant [muscle × precue [*F*(8,120) = 9.69, *p *< 0.001; muscle × time *F*(6,90) = 3.75, *p *< 0.05; muscle × precue × time [*F*(24,360) = 4.33, *p *< 0.01]. The main effect of muscle indicated that the MEP amplitudes differed between muscles, as can be seen in Figure 7. As expected, TMS over the optimal position for evoking thumb movements resulted in largest MEPs in the thumb muscle (APB), smaller MEPs in the index finger muscle (FDI), and smallest MEPs in the wrist muscle (FCR). As the three-way interaction indicated that the modulation of MEP amplitudes by precue and time differed in the three muscles, this effect was further specified with a separate two-way ANOVA (precue × time) for each muscle.

MEP amplitudes of the APB were significantly modulated by the precue [*F*(4,60) = 13.87, *p *< 0.001]. The effect of time was not significant [*F*(3,45) = 2.77, n.s.], but there was significant precue × time interaction [*F*(12,180) = 5.28, *p *< 0.01]. This interaction pointed to a differential effect of the precue across the four time points. To determine at which time points the precue differentially modulated the MEP amplitude, separate one-way ANOVAs with the factor precue were conducted on the MEP amplitudes at each of the four stimulation times. This showed that at -900 ms (baseline) and -300 ms the MEP amplitudes were not different for the five precues [*F*(4,60) = 0.48 and *F*(4,60) = 2.09 respectively, both n.s.]. However, at -100 ms and 250 ms, there was a significant effect of precue on the MEP amplitudes [*F*(4,60) = 8.83, *p *< 0.001; *F*(4,60) = 11.32, *p *< 0.001; respectively]. Post-hoc comparisons revealed that at -100 ms and at 250 ms, the MEP amplitudes in the conditions with 180°, 225°, 270° precues were significantly smaller than in conditions with 90° and 135° precues (pairwise significance levels detailed in Table 1).

**Table 1:** Post-hoc comparisons of APB MEP amplitudes

Pairwise comparison	-100 ms	250 ms
90° vs. 180°	*p *< 0.05	*p *< 0.01
90° vs. 225°	*p *< 0.01	*p *< 0.01
90° vs. 270°	*p *< 0.01	*p *< 0.05
135° vs. 180°	n.s.	*p *< 0.001
135° vs. 225°	*p *< 0.01	*p *< 0.001
135° vs. 270°	*p *< 0.01	*p *< 0.01

The MEP amplitudes of the FDI were significantly modulated by time [*F*(3,45) = 20.20, *p *< 0.001], but not by precue [*F*(4,60) = 0.83, n.s.]. The precue × time interaction was not significant [*F*(12,180) = 2.02, n.s.]. Post-hoc comparisons showed that FDI MEP amplitudes progressively decreased from -900 (baseline) through -100 ms, and remained unchanged from -100 through 250 ms (pairwise significance levels detailed in Figure 7B).

The MEP amplitudes of the FCR were not different between precues [*F*(4,60) = 0.57, n.s.], nor stimulation times [*F*(3,45) = 2.89, n.s.], and that there was no interaction between these factors [*F*(12,180) = 0.75, n.s.] (Figure 7C).

In short, the MEP amplitudes from the thumb and the finger muscles were modulated during the preparatory interval and the response interval. For the finger muscle this effect was similar regardless of the direction conveyed by the precue. For the thumb muscle the effect of the precue depended on the time point in the trial. There was a significant precue effect during the preparatory interval, as well as during the response interval. The MEP amplitudes in the wrist muscle remained unchanged.

### Background muscle activity

The ANOVAs on the pretrigger EMG RMS amplitudes showed that there was no modulation of background muscle activity over time in the APB [*F*(3,45) = 1.77, n.s.], in the FDI [*F*(3,45) = 1.40, n.s.], and in the FCR [*F*(3,45) = 0.59, n.s.]. Thus, the observed changes in TMS-evoked movements or MEP amplitudes can not be explained by changes in background muscle activity.

## Discussion

The aim of this study was to examine the effects of prior knowledge about the direction of an impending thumb movement on both the thumb movement representation (reflected by TMS-evoked movements) and on corticospinal excitability (measured as changes in MEP amplitudes). Our data show that during the preparation of a directed thumb movement progressive changes in TMS-evoked movements occur. We found that the direction of TMS-evoked movements became increasingly unpredictable but contrary to our expectation, the TMS-evoked movements did not change towards the precued direction (Figure 6). However, the MEP amplitudes did show a precue specific modulation of corticospinal excitability in the thumb muscle while other muscles only displayed a-specific excitability decreases over time or no modulation at all.

In the early-precue task, TMS was given in 78% of the trials. Since supra-threshold TMS evoked a thumb movement itself, the TMS pulse can be conceptualized as a perturbation that interferes with preparatory processes. To control for a direct interference of TMS on voluntary movement and reaction time, we regarded only data of trials without TMS. On a more general level, some kind of strategic compensation for expected TMS perturbations might have influenced the subjects in the early-precue task. Nevertheless, the behavioural data show that this did not abolish the intended experimental manipulations and that the subjects' performance conformed to the task's requirements. The actual direction of the voluntary thumb movements closely matched the direction instructed by the precue. Moreover, the five precues elicited voluntary thumb movements in five distinct directions. Thus, the precue induced the programming of a thumb movement in the corresponding direction. When the required movement direction was precued early in the preparatory interval reaction time was much faster than when the direction was precued at the end of the interval, in agreement with previous work [2,3,33]. This strongly suggests that parts of the thumb movement were programmed before the response signal occurred.

The thumb movement representation underwent progressive changes during motor preparation. The increased variability of TMS-evoked movements became apparent already during the preparatory interval. It is tempting to assume that these changes were evoked by the directional information conveyed by the precue. However, as there was no indication that the TMS-evoked movements turned towards the precued direction, we cannot conclude whether or not these changes were due to implementation of the direction of the thumb movement (Figure 6). That said, the MEP data revealed that the directional information of the precue was integrated into the preparation on the level of the motor output system, because it affected corticospinal excitability in a muscle, time, and precue specific manner (Figure 7). The electrodes to measure the EMG of the thumb were placed above the APB, which is the predominant muscle for abduction movements. Directly besides the APB lies the flexor pollicis brevis, the muscle responsible for thumb flexion. When the precue instructed an abduction/flexion movement the MEP amplitudes from the thumb musculature were significantly lower than when the precue instructed an abduction/extension. In addition, this effect was present only at 100 ms before and 250 ms after the response signal and it was specific to the thumb musculature. The MEP amplitudes of the index finger muscle, not primarily involved in thumb movements, progressively decreased during the preparatory interval, but this decrease was not different between precues.

In accordance with previous work [26-29,31] our data indicate that corticospinal inhibition is a prominent aspect of motor preparation. Both the movement agonist and a neighbouring muscle showed decreases in corticospinal excitability during the preparatory delay. It is possible that a non-specific activation by the warning signal had initially increased MEP amplitudes and that they subsequently returned to resting levels, because baseline measures were obtained after the warning signal was presented. More likely, the inhibitory effects in our data are partly related to the timing of the early precue. Corticospinal inhibition is typically pronounced with relatively short intervals of about 500 ms and was not expected to play a major role in our task. Originally, we conceptualized the preparatory interval of our tasks as the interval between the neutral warning signal and the response signal, which was 1200 ms. However, the occurrence of the early precue halfway the preparatory interval provided an additional temporal cue (i.e. warning signal) to the subjects. As such, the early precue gave rise to a new sub-interval of 600 ms. Such intervals are prone to corticospinal inhibition [25-29]. Recent work suggests that motor inhibition plays a role in withholding general motor activation caused by warning signals [34]. Another aspect that may have boosted motor inhibition is the inclusion of no-go trials. The no-go trials were included to prevent premature (i.e. before the response signal) response tendencies, which is important when investigating purely preparatory processes. However, the no-go trials introduced uncertainty about the actual requirement of a movement. As a consequence, any response tendencies had to be suppressed until the subject was certain about the need for a response. Such a 'corticospinal braking mechanism' may have caused the observed reduction of MEP amplitudes across the preparatory interval [23,26,31]. These considerations should be taken into account in future TMS studies on motor preparation. Experimental designs without catch trials and/or using variable or relatively long preparatory intervals may significantly reduce inhibitory tendencies, although recent data suggests that long or variable intervals not necessarily eliminate motor inhibition [23,31].

In an earlier study, Sommer and colleagues showed that TMS-evoked movements follow the intended direction during the end of the response interval [15]. What can explain that in the response interval of our experiment the TMS-evoked movements did not correspond to the intended movement direction? In the experiment of Sommer et al. the TMS-evoked movements did not follow the intended direction until 90 ms before movement onset. This corresponds to the time at which the pre-movement increase in corticospinal excitability starts [15-21]. A sharp increase in MEP amplitudes in that period would suggest the release of the corticospinal brake [35]. The reaction times and MEP data in our study indicate that the pre-movement excitability increase had not been initiated at the latest stimulation time (250 ms after response signal). It has been suggested that motor inhibition secures the development of a motor plan without leading to premature output [26,30]. Hence we believe that in our experiment the movement direction was integrated into the motor plan, but the program could not be released (either by TMS or voluntarily) due to a superimposed braking mechanism. After this brake had been withdrawn, the involved muscles became facilitated in correspondence to the parameters of the forthcoming movement.

## Conclusion

We have shown that during preparation of a voluntary thumb movement, corticospinal output probed by TMS-evoked movements displays a progressive modulation. TMS-evoked movements increasingly deviated from the baseline direction, but did not turn towards the precued direction. The modulation of TMS-evoked movements over the preparatory interval was accompanied by inhibitory changes in corticospinal excitability that were muscle specific and depended on the prepared direction (implying that directional information was integrated). Earlier studies have shown that shortly before movement onset, when corticospinal excitability increases sharply, TMS-evoked movements do turn to the intended direction. Taken together, this suggests that during preparation, a corticospinal braking mechanism is active to counteract concurrent facilitatory processes. This inhibition ceases just before movement onset, releasing the intended movement.

## Methods

### Subjects

Sixteen healthy volunteers (11 female and 5 male) with a mean age of 24 years (range 20–30) participated in the experiment. All were right-handed (mean Oldfield [36] handedness score of 93, range 60–100) and had normal or corrected-to-normal visual acuity. Subjects were screened for any history of neurologic illness or neurosurgery and for any metal or electronic implants. All subjects gave written informed consent prior to the experiment. The experimental procedures were in accordance with the declaration of Helsinki and approved by the local ethics committee of the Radboud University Nijmegen.

### Procedure and task

**Figure 8:**
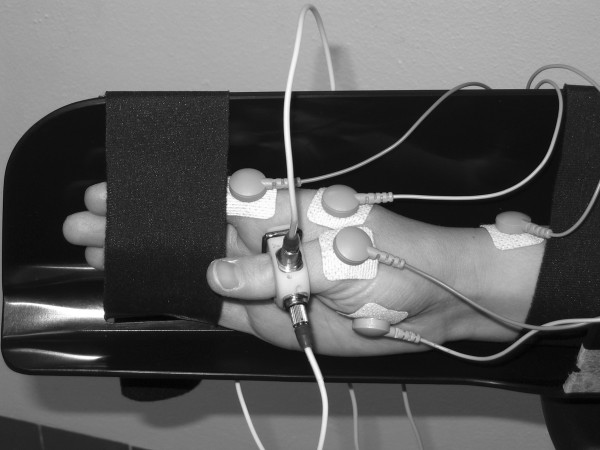
**Experimental setup**. The right arm was fixed with Velcro straps. Thumb movements were measured by two miniature uni-axial accelerometers that were mounted on the proximal phalanx of the thumb, in orthogonal planes. Electromyographic activity from the thumb muscle (abductor pollicis brevis), index finger muscle (first dorsal interosseus), and wrist muscle (flexor carpi radialis; not visible on photo) was recorded using adhesive Ag/AgCl surface electrodes.

Subjects were seated in a comfortable chair, in front of a 15 inch computer screen (distance ~75 cm). The subject's right forearm, wrist, and fingers 2–5 were immobilised in a tight U-shaped cast with the elbow flexed 90 degrees and the forearm semi-pronated. The thumb was left entirely free to move (see Figure 8). In this setup, the axes of thumb abduction/adduction and of thumb flexion/extension were close to the horizontal and vertical space axes, respectively.

The experimental tasks required subjects to move the thumb of their right hand in response to a series of visual signals, as quickly and as accurately as possible. Figure 1 gives a schematic representation of the time course of a typical trial. In each trial, three signals (with a visual extent of ~0.5°) were presented in the centre of a circle (diameter ~8° of visual angle) that remained on the computer screen throughout the experiment. First, a neutral warning signal (blue square) marked the beginning of a new trial. After a 1200 ms delay this warning signal was replaced by a response signal instructing the subject to move the thumb (go trial; green square; 80% probability) or to withhold a response (no-go trial; red square; 20% probability). The required direction of each thumb movement was precued during the interval between warning and response signal, by a blue arrow that was briefly flashed (100 ms) at the position of the warning signal. The five possible directions (90°, 135°, 180°, 225°, and 270°) were always tick-marked on the circle. One of these five movement directions was precued in each trial. There were two task variants, which only differed in the timing of the directional precue. In the early-precue task the direction was specified 600 ms before the response signal, whereas in the late-precue task it was specified 100 ms before the response signal. Subjects were instructed to completely relax their arm muscles during the period preceding the response signal and to respond in go trials only. After go trials, a marker was plotted on the circle to show the direction of the movement just executed. After no-go trials, a centrally presented green or red square informed the subject whether the response was correctly withheld or not.

Before performing the experimental tasks subjects were trained extensively. The aim of the training was to familiarise the subjects with the required stimulus-response associations and to practice complete muscle relaxation whenever movement was not appropriate. Furthermore, subjects who had no prior experience with TMS were familiarised with the technique. A large part of the training was completed in a separate session one or two days before the test session. In the first session the subjects initially performed a short warm-up task. This task was identical to the late-precue task except that the precued directions on subsequent trials were arranged orderly, in an anti-clockwise fashion (90°, 135°, 180°, 225°, 270°, 90°, etc. etc.). Subjects completed at least two blocks of 30 trials. If movements were inaccurate, additional blocks were presented until the experimenters deemed performance to be adequate. Next, the subjects performed three blocks (40 trials each) of the early-precue task and three blocks of the late-precue task (counterbalanced across subjects). Auditory feedback of electric muscle activity was provided continuously during all three tasks. Finally, subjects unfamiliar with TMS were introduced to the technique. In the second session the subjects initially performed the warm-up task while auditory feedback of their muscle activity was provided. Again, at least two blocks of 30 trials were completed. If movements were inaccurate or excessive muscle activity was present prior to movement initiation, additional blocks were presented until performance was deemed adequate. After their movement threshold was determined (see below), the subjects practiced 20 trials of the subsequent early-precue task, which included the application of TMS pulses during task performance. Subjects were asked to ignore any possible interference of TMS and perform the task to their best ability. The experimental tasks were carried out in the final part of the second session. The subjects completed nine blocks of the early-precue task followed by two blocks of the late-precue task. According to our hypothesis, reaction times in the late-precue task were expected to be longer than in the early-precue task. The late-precue task was always performed after the early-precue task. In this way, if a training effect would occur, it would only lead to an underestimation of the precue effect, as the effects would have opposite directions. In a random 280 (~78%) out of the 360 early-precue trials a single TMS pulse was applied at either 900 ms (40 trials), 300 ms (80 trials), or 100 ms (80 trials) before, or 250 ms (80 trials) after the response signal. No TMS was applied in the late-precue task. As TMS frequently evoked involuntary thumb movements, feedback was omitted after TMS trials. Each block consisted of 40 trials, resulting in 360 early-precue trials, (280 with and 80 without TMS) and 80 late-precue trials.

### Transcranial magnetic stimulation

TMS was delivered using a figure-of-eight shaped coil (diameter of each wing 70 mm) connected to a Magstim 2002 stimulator (Magstim Company, Whitland, United Kingdom). The coil was positioned tangentially on the left hemiscalp with its handle pointing backward at an angle of about 45 degrees from the midsagittal axis. First, the optimal position for evoking isolated thumb movements was identified. At this position the movement threshold was determined. Movement threshold was defined as the lowest stimulator output evoking a thumb acceleration of ≥ 0.9 ms^-2^ in at least five out of eight successive stimulations. Stimulation intensity was set slightly above this threshold, on average 49 (± 10) % of maximum stimulator output. Coil position was monitored continuously (BrainSight TMS, Rogue Research, Montreal, Canada) and adjusted whenever its distance to the optimal stimulation position exceeded 5 mm.

### Data acquisition

**Figure 9:**
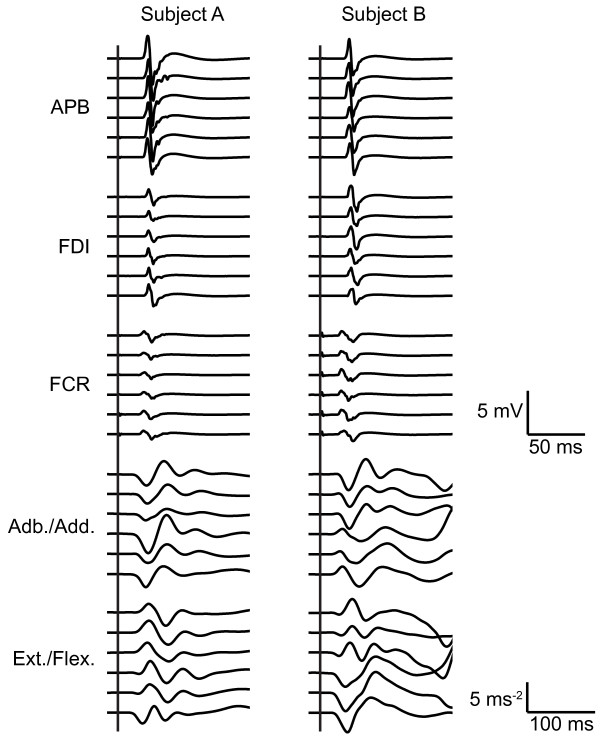
**Example EMG and accelerometer traces**. Example traces of responses to TMS, from two of the subjects. Of each of the two subjects, six arbitrary TMS-trials were selected and the EMG and accelerometer signals recorded in those trials are plotted. The upper six sets are EMG traces; the lower four sets are accelerometer traces. The vertical lines mark the time when the TMS pulse was applied.

Thumb movements were recorded by two miniature uni-axial accelerometers (Model 256–100, sensitivity 10 mV/ms^-2^; Endevco Corp., San Juan Capistrano, CA) that were mounted on the proximal phalanx of the thumb in orthogonal planes to detect acceleration in the abduction/adduction and extension/flexion axes. Accelerometer signals were conditioned with a gain of 10 (Model 4416B signal conditioner, Endevco Corp.). EMG from the thumb muscle (electrodes over the abductor pollicis brevis 'APB'), index finger muscle (electrodes over first dorsal interosseus 'FDI'), and the wrist muscles (electrodes over flexor carpi radialis 'FCR') of the right hand was recorded using adhesive Ag/AgCl surface electrodes (Kendall-LTP, Chicopee, MA). Electrodes were placed in "belly-tendon" arrangements, following standard skin preparation. EMG signals were amplified with a gain of 250 using an Ekida amplifier (Ekida GmbH, Helmstadt, Germany). Accelerometer and EMG signals were anti-aliasing filtered (1 kHz cut-off), then digitised at a rate of 5 kHz (acceleration resolution 0.15 ms^-2^/bit, voltage resolution 0.61 μV/bit) using Spike2 software and a Power 1401 A/D converter (Cambridge Electronic Design, Cambridge, United Kingdom). Figure 9 shows some example traces of TMS responses in EMG and accelerometer signals.

### Data processing and analysis

Data were processed off-line using MATLAB (MathWorks, Natick, MA). Acceleration and EMG data were digitally filtered (low-pass 100 Hz, band-pass 10–500 Hz, respectively) and segmented into epochs running from 1200 ms before to 800 ms after each response signal. The two accelerometer signals were converted into polar coordinate (magnitude-angle) time series. A peak detection algorithm was applied to the magnitude of this signal to determine onsets and corresponding directions of voluntary and of TMS-evoked movements.

Reaction time (RT) was defined as the latency between the response signal and the first peak of acceleration. Because TMS may influence RT [23,37-40] only trials without TMS were used for the analyses of voluntary movements (with the exception of the analysis that assessed the effect of TMS on RT; see below). Per subject, trials with an RT of more then 2.5 standard deviations from the mean RT were discarded from all analyses. The RT of the early- and late-precue conditions was compared with a two-tailed paired-samples *t*-test. The effect of the precue on the direction of the subsequent voluntary thumb movement was analysed with a one-way repeated-measures ANOVA with the within-subjects factor precue (90°, 135°, 180°, 225°, 270°) and the first-peak acceleration angle as dependent variable. To assess the contribution of the three muscles to the different movements, we calculated the average root mean square (RMS) amplitude during the first 150 ms of the EMG bursts associated with the voluntary movements.

We performed an additional analysis on the RTs to assess the effect of a TMS perturbation on motor preparation. For each trial in which TMS was applied during the preparatory interval (at -900 ms, -300 ms, or -100 ms) the RT was determined. Trials with an RT of more than 2.5 standard deviations from the mean RT were discarded. The remaining RTs were compared to the RT from early-precue trials without TMS using a one-way ANOVA with the within-subjects factor time (no TMS, -900 ms, -300 ms, -100 ms).

The required direction of the upcoming movement was precued not before 600 ms prior to the response signal. Therefore the period between 1200 and 600 ms prior to the response signal was termed the baseline interval. Responses to TMS given in this interval (i.e. stimulation time -900 ms) were considered as an individual baseline for analyses of TMS-evoked movements, MEPs, and pretrigger RMS amplitudes.

TMS-evoked movements should have more-or-less constant latencies, because these depend mechanistically on the conduction time of the nervous pathway. Consequently, trials where the latency of the first-peak acceleration of TMS-evoked movements deviated more than 10 ms from the mode across all latencies were discarded from all analyses. To assess whether the precue influenced the direction of subsequent TMS-evoked movements, we defined a "baseline zone". This was a window of ± 30° centred on the average direction of TMS-evoked movements at baseline. We assessed whether there was a temporal modulation of the thumb movement representation by calculating the proportion of TMS-evoked movements that fell outside this baseline zone, at each stimulation time. These proportions were then submitted to a one-way repeated-measures ANOVA with time as a within-subjects factor (-900 ms, -600 ms, -100 ms, 250 ms). A further analysis was conducted to elucidate whether any changes in the thumb movement representation reflected the direction that was precued. Therefore, we determined the proportion of TMS-evoked movements that fell within a ± 30° window centred on the direction that had been precued (i.e. "precued target zone"). Analogous to the previous analysis, the proportion of TMS-evoked movements within the precued target zone was analysed with a one-way repeated-measures ANOVA with the within-subjects factor time (-900 ms, -600 ms, -100 ms, 250 ms).

Corticospinal excitability was assessed by the peak-to-peak MEP amplitude between 10 and 50 ms after the TMS trigger. To make sure the target muscles were at rest during the critical period of each trial, a trial was discarded from all analyses if voluntary EMG during the 200 ms preceding the TMS pulse or preceding the response signal exceeded 50 μV. In addition, the EMG RMS amplitudes 100 ms prior to TMS were calculated. To reduce between-subject variability, the MEP and pretrigger RMS amplitudes were normalised to the average MEP or average pretrigger RMS amplitude (respectively) across the three muscles measured at baseline (a value of 1 was assigned and all other values expressed relative to this value). The normalised MEP amplitudes were initially submitted to a three-way repeated-measures ANOVA with within-subjects factors muscle (APB, FDI, FCR), precue (90°, 135°, 180°, 225°, 270°) and time (-900 ms -600 ms, -100 ms, 250 ms). Significant interactions were further specified with separate two- and one-way ANOVAs. To assess whether preliminary muscle activation could explain any temporal modulation of TMS-evoked movements or MEP amplitudes, one-way ANOVAs with the within-subjects factor time were also conducted on the normalised pretrigger RMS amplitudes of each muscle.

Degrees of freedom were adjusted with the Greenhouse-Geisser epsilon if the sphericity assumption was not met, but for statistical interpretation uncorrected degrees of freedom are reported. Statistical significance was set at the 0.05 level. Significant effects in the omnibus tests were taken as justification for further specification by post-hoc Fisher's LSD tests. Unless stated otherwise, data are presented as mean ± standard error of mean (*SE*).

## Competing interests

The authors declare that they have no competing interests.

## Authors' contributions

GvE, WDS, and SO designed the experiment. GvE and WDS acquired the data, and performed the analyses. GvE drafted the manuscript. All authors participated in the interpretation of the data, in discussions and general conclusions, in revising the manuscript, and read and approved the final manuscript.
